# Characterization of MicroRNAs from *Orientobilharzia turkestanicum,* a Neglected Blood Fluke of Human and Animal Health Significance

**DOI:** 10.1371/journal.pone.0047001

**Published:** 2012-10-10

**Authors:** Chun-Ren Wang, Min-Jun Xu, Jing-Hua Fu, Alasdair J. Nisbet, Qiao-Cheng Chang, Dong-Hui Zhou, Si-Yang Huang, Feng-Cai Zou, Xing-Quan Zhu

**Affiliations:** 1 State Key Laboratory of Veterinary Etiological Biology, Key Laboratory of Veterinary Parasitology of Gansu Province, Lanzhou Veterinary Research Institute, Chinese Academy of Agricultural Sciences, Lanzhou, Gansu Province, People's Republic of China; 2 College of Animal Science and Veterinary Medicine, Heilongjiang Bayi Agricultural University, Daqing, Heilongjiang Province, People's Republic of China; 3 College of Animal Science, South China Agricultural University, Guangzhou, Guangdong Province, People's Republic of China; 4 Parasitology Division, Moredun Research Institute, Pentlands Science Park, Midlothian, Scotland, United Kingdom; 5 College of Animal Science and Technology, Yunnan Agricultural University, Kunming, Yunnan Province, People's Republic of China; Ghent University, Belgium

## Abstract

The neglected blood flukes *Orientobilharzia* spp. belonging to the Platyhelminthes, infect animals in a number of countries of the world, and cause cercarial dermatitis in humans, as well as significant diseases and even death in economically-important animals. MicroRNAs (miRNAs) are now considered to be a key mechanism of gene regulation. Herein, we investigated the global miRNA expression profile of adult *O. turkestanicum* using next-generation sequencing technology and real-time quantitative PCR, to gain further information on the role of these molecules in host invasion and the parasitic lifestyle of this species. A total of 13.48 million high quality reads were obtained out of 13.78 million raw sequencing reads, with 828 expressed miRNAs identified. Phylogenetic analysis showed that the miRNAs of *O. turkestanicum* were still rapidly evolving and there was a “directed mutation” pattern compared with that of other species. Target mRNAs were successfully predicted to 518 miRNAs. These targets included energy metabolism, transcription initiation factors, signal transduction, growth factor receptors. miRNAs targeting egg proteins, including major egg antigen p40, and heat shock proteins were also found. Enrichment analysis indicated enrichment for mRNAs involved in catalytic, binding, transcription regulators and translation regulators. The present study represented the first large-scale characterization of *O. turkestanicum* miRNAs, which provides novel resources for better understanding the complex biology of this zoonotic parasite, which, in turn, has implications for the effective control of the disease it causes.

## Introduction

*Orientobilharzia* spp. are schistosomes that cause orientobilharziasis in a wide range of animals including cattle, sheep, goats and other mammals [1]. They live in the portal or intestinal veins of the infected animals and cause emaciation, anemia and diarrhea, with sloughed mucosal material and blood in the host feces. Infection may lead to acyesis or abortion in females and inhibits the growth of young animals. *Orientobilharzia* spp. are distributed across a number of countries including India, Mongolia, Russia, Iran, Turkey and China [2], [3], causing severe infections and significant economic losses in production animals in these areas. For example, following the flooding of a river in China in 1998, infection of goats with *O. turkestanicum* caused a 40% fatality rate in the animals grazing on the flood plain [1]. Similarly, a 10% fatality rate was reported from another goat farm in Daqing in China in 2006, with more than 30,000 adult *O. turkestanicum* found in the intestinal veins of one infected goat [1].

Importantly, from a human health perspective, *Orientobilharzia* spp. have been confirmed as zoonotic agents, causing cercarial dermatitis in humans with acute inflammatory responses characterized by pruritus and skin papulation. As the disease develops, abrasions and skin infections may occur as a result of scratching. Cercarial dermatitis has been reported from many localities including South Africa, USA, United Kingdom, mainland Europe, India, Australia, China and elsewhere in Asia [1], [4]–[13]. However, despite the significant economic and social impact of the parasites, there has been a paucity of published studies relating to *Orientobilharzia* biology, genomics and novel control technologies compared with other pathogens causing cercarial dermatitis, such as *Trichobilharzia* spp. [11], [14]–[16].

microRNAs (miRNAs) are non-coding RNAs that regulate gene expression at the post-transcriptional level, resulting in post-transcriptional repression, cleavage or destabilization and are now considered as key mechanism of gene regulation. Furthermore, miRNAs are crucial for the regulation of the complex life cycles of parasites, allowing them to respond to environmental and developmental signals [17], [18]. In order to obtain a comprehensive understanding of *Orientobilharzia* spp. gene regulation, and thus inform novel control technologies, here we have investigated the microRNA expression profile of *Orientobilharzia* spp. by using *O. turkestanicum* adults as a representative stage and species. Because of their similarities in invasion mechanisms and parasitic life cycles, the investigation of miRNA profiles of *Orientobilharzia* spp. will inform studies on other schistosomes, such as *Trichobilharzia* spp., *Schistosoma spindale* and *Bilharziella* sp.

## Materials and Methods

### Ethics Statement

The sheep from which *O. turkestanicum* adults were collected were taken from a local abattoir (Longfeng Slaughterhouse, Daqing City, Heilongjiang Province, China). These animals were being processed as part of the normal work of the abattoir.

### Parasite Material

Adults of *O. turkestanicum* were collected from the portal and mesenteric veins of a sheep with naturally acquired infection from the Longfeng Slaughterhouse, Daqing City, Heilongjiang Province, China in March 2011. *O. turkestanicum* infection in sheep in this region occurs from July to September, and matured two months later. Therefore, harvested flukes in March were all matured adults in the present study, with female adults being 7–9 mm and male adults being 5–8 mm in length. Furthermore, to confirm that they were at adult stage, worms were randomly selected and stained with hematoxylin staining solution or carmine staining solution. For female adults, the uterus contains one egg at a time which has a terminal spine with a short appendage at one end and has no operculum; For male adult, testes can be found as particles with the number of approximately 80 [19]. Fifty parasites were washed extensively in physiological saline and then stored at −80°C until use. Species identity was confirmed by internal transcribed spacer (ITS-1 and ITS-2) analyses [20] and by sequencing of the mitochondrial cytochrome *c* oxidase subunit 1 (*cox*1) and nicotinamide adenine dinucleotide dehydrogenase subunit 1 (*nad*1) genes [21].

### Total RNA and Small RNA Isolation

Total RNA was extracted using TRIzol Reagent according to the manufacturer’s protocol (Invitrogen Co. Ltd). Small RNA isolation was performed as described previously [22]. Briefly, 10 µg total RNA and a Novex 15% TBE-Urea gel were used for the isolation of RNA fragments of 20–35 bases in length. These fragments were then ligated to 5′ and 3′ adaptors (Illumina), reverse transcribed and purified using a 6% TBE PAGE gel. All gels and kits for small RNA purification and amplification were purchased from Invitrogen Co. Ltd.

### High-throughput Sequencing and Computational Analysis

Samples were sequenced, as described previously, using a Solexa (Illumina) sequencer [23]. Adaptors and low quality reads were first removed from the raw dataset. Non-coding RNA, including rRNA, tRNA, snRNA, snoRNA, and other non-coding RNA were removed by searching against the GenBank and Rfam databases (version 10.1) (http://rfam.sanger.ac.uk/). Repetitive sequences were removed using RepeatMasker (http://www.repeatmasker.org). The remaining reads were searched against the Sanger miRBase (version 17.0) to identify conserved miRNAs with mismatches ≤2 [24], [25]. Because no publically-available genome is currently accessible for *Orientobilharzia* spp., the genome of the related schistosome, *S*. *japonicum,* (http://lifecenter.sgst.cn/schistosoma/cn/schistosomaDispatch.do?disName=static) was used as a reference genome, using the Short Oligo nucleotide Analysis Package (SOAP) software to map reads from *O. turkestanicum* [26], [27]. The software Mfold (http://www.bioinfo.rpi.edu/applications/mfold) was used for the prediction of novel miRNAs with free energy hybridization of the precursor (hairpin) lower than -18 kcal/mol [23].

The cDNA data with annotation of blood flukes were downloaded from the http://www.chgc.sh.cn/japonicum/Resources.html. Potential targets of known miRNAs were predicated with RNAhybrid software against the datasets above under default parameters. To reduce false-positive result, two extra parameters were performed to the analyzed result: 1) the △△G was set as lower than -25 kcal/mol; 2) P-value was set as ≧ 0.01. The Gene Ontology (GO, http://www.geneontology.org/) database was used for functional analysis of predicted target.

### Analysis of Novel miRNA Expression

Novel miRNAs were analyzed using a modified stem-loop real-time RT-PCR (ABI PRISM® 7300 Sequence Detection System) as described previously [23], [28]. All of the primers were synthesized by Shenggong Co, Ltd., China. Real-time quantitative PCR was performed in a 20 µl reaction. All reactions were carried out in triplicate. Synthetic *lin-4* was used as the endogenous control [29]. The amplification cycle conditions were as follows: 95°C 5 min, followed by 30 cycles of 95°C for 15 s, 65°C for 15 s, and 72°C for 32 s. The quantification of each miRNA relative to the alpha-tubulin transcript was calculated using the equation: N = 2^−ΔCt^, ΔCt = Ct_miRNA_−Ct_tubulin_ [30], [31].

## Results

### Profile Characteristics of Short RNAs from *O. turkestanicum*

**Figure 1:**
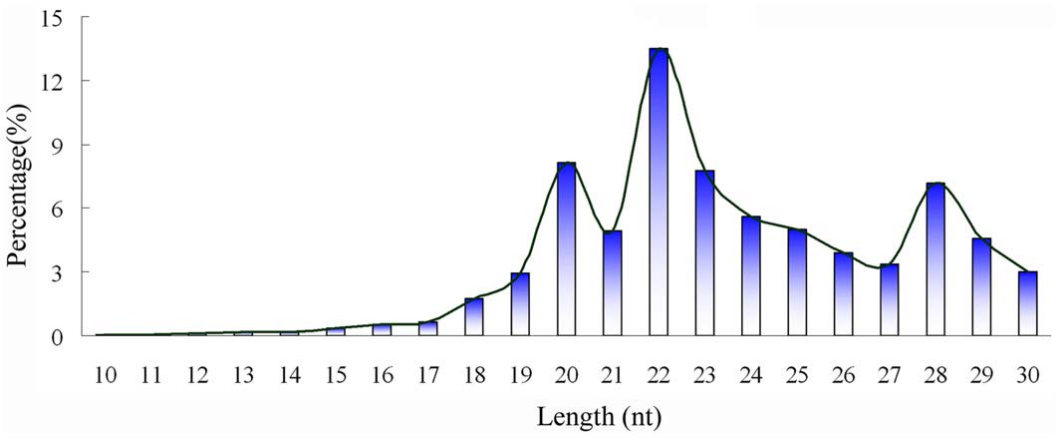
Length distribution analysis of small RNAs of *Orientobilharzia turkestanicum*.

13.78 million raw reads were obtained from total RNA of adult *O. turkestanicum* by Solexa deep sequencing, 13.48 million of which were of “high quality”. After removing adaptors and poly-A sequences, there were 12,867,087 reads (95.45% of the high quality reads) remaining and these were marked as “clean reads” for further bioinformatic analysis (Table 1). Length distribution analysis showed that most of the clean reads were 22 nt in length, followed by those of 20 and 23 nt (Figure 1).

When searched against public databases, non-coding RNA including rRNA, snRNA, snoRNA, repeats, exons and introns were identified (Table 1). Those representing exons and introns accounted for a very small percentage of the clean reads, which indicated high integrity of the RNA in the sample. On the other hand, it was found that “repeat” sequences represented a relatively high percentage (when compared with other parasite species) of total sRNA (0.07%) and unique sRNA (0.38%) sequences from *O. turkestanicum*. In other parasites for which data were examined, repeat reads occurred with a 10-fold lower frequency than this [e.g. *Clonorchis sinensis* (0.02%) [23], *Fasciola gigantica* (0.01%, unpublished data), *F. hepatica* (0.01%, unpublished data), five strains of *Toxoplasma gondii* (0.00%, unpublished data)].

**Table 1:** Annotation of short RNA clean reads of *Orientobilharzia turkestanicum*.

	Unique siRNA^a^ (%)	Total siRNA^b^ (%)
**Total**	1372184	12867087
**exon_antisense**	803 (0.06)	3052 (0.02)
**exon_sense**	4469 (0.33)	130253 (1.01)
**intron_antisense**	1950 (0.14)	10321 (0.08)
**intron_sense**	2240 (0.16)	59492 (0.46)
**miRNA**	70435 (5.13)	2225558 (17.30)
**rRNA**	281255 (20.50)	7398623 (57.50)
**repeat**	5150 (0.38)	8407 (0.07)
**snRNA**	3015 (0.22)	6839 (0.05)
**snoRNA**	373 (0.03)	661 (0.01)
**tRNA**	23266 (1.70)	246278 (1.91)
**unann**	979228 (71.36)	2777603 (21.59)

*Note*: **^a^**Unique siRNA indicates sequenced reads without redundancy, which means that one read had only one copy in the dataset; ^b^Total siRNA indicates all of the sequenced reads, which means that one read might have plenty of copies in the dataset.

### Analysis of miRNA Profiles of *O. turkestanicum*

After removing non-coding RNAs, a total of 2,225,558 (17.30%) reads were searched against the Sanger miRBase. Finally, 828 conserved miRNAs were identified which composed the miRNA expression profile of the parasite, indicating a high level of redundancy in miRNA expression in *O. turkestanicum*. There were 8 miRNA sequences with copy numbers of 100,000 or higher, including miR-2206-3p, miR-4006f-5p, miR-1692, miR-4208-3p and 4 members of the miRNA-1 family. The highest copy number was found for miR-1c with 571, 676 copies, followed by miR-1b with a copy number of 547,526 and miR-2206-3p with a copy number of 531,894. There were 190 miRNAs with a copy number <3.

Three distinct families were found within the miRNA expression profile of *O. turkestanicum*; miR-1, let-7 and miR-2. The miR-1 family was distinctive for the high number of copies of its members, with a total of 1,752,199 copies. Five members of this family were represented within the miRNA dataset: miR-1, miR-1a, miR-1b, miR-1c, and miR-1*. The let-7 family was the miRNA family with the highest number of members (14) represented including let-7a–k, let-7 itself and a star sequence, let-7b*. Among the let-7 family, let-7f was the most highly represented with 48,638 copies, followed by let-7 with 19,204 copies. The miR-2 family was the second most highly represented family with 6 members from miR-2a to miR-2f. There was no large copy number bias for any member of the miR-2 though the most abundant was miR-2a with 3,790 copies.

**Figure 2:**
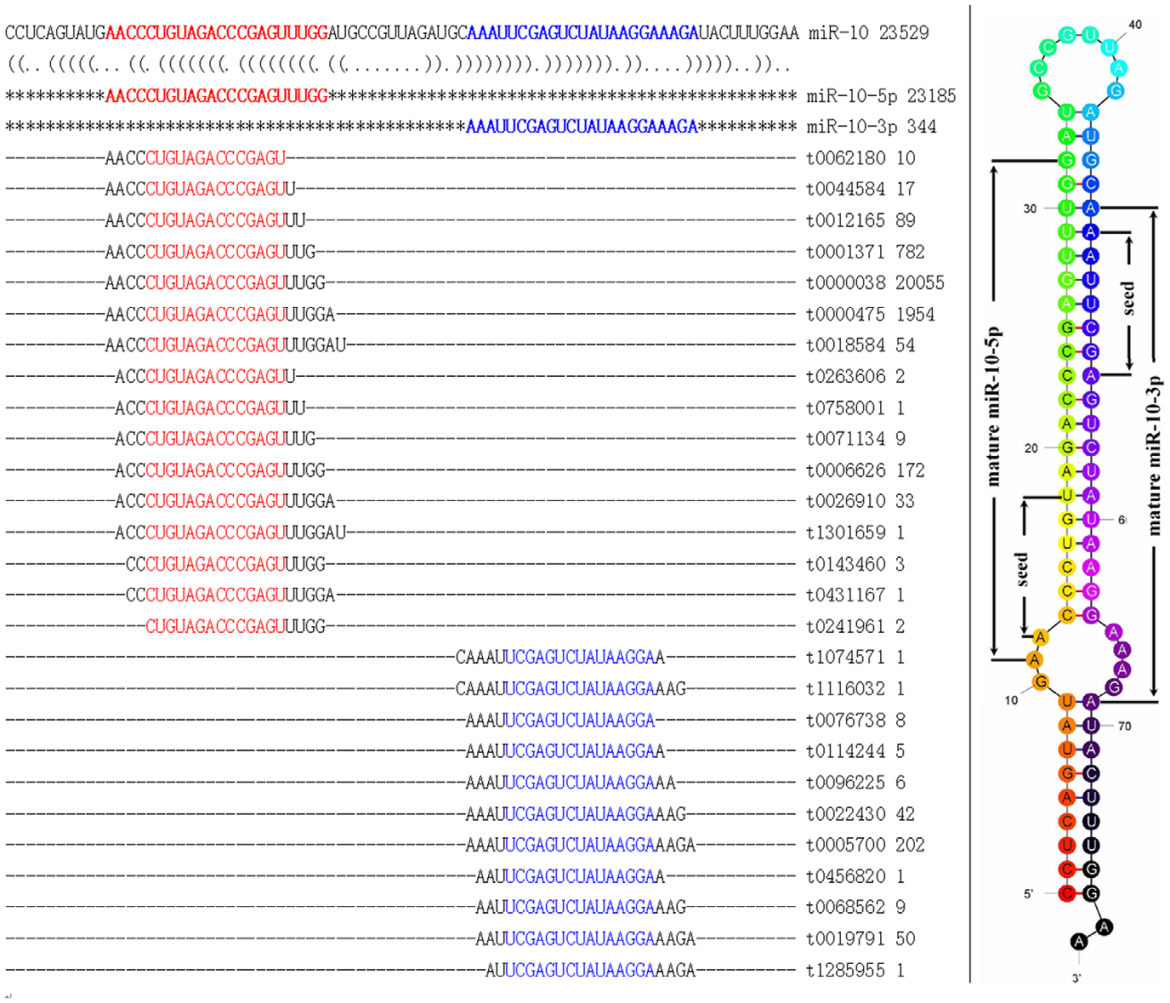
Precursor, canonical sequence with variants and secondary structure of miR-10-5p of *Orientobilharzia turkestanicum*. Left: BLAST alignment of miR-10-5p canonical sequence with variants. Red and blue show the miR-10-5p and its star sequence and the conserved parts of the variants. Right: secondary structure of the miR-10 precursor with the seed region indicated. Star sequence is reads with the highest copy number that matched with 3′ ends of precursors; others matched were regarded as variants.

There were 34 miRNAs with star sequences, including miR-1, miR-125b, miR-10-5p, miR-214 and miR-745. In most cases, the copy number of the mature miRNA was higher than its associated star sequence. For miR-10-5p, 23,185 copies were present, while its star sequence miR-10* (also called miR-10-3p) was represented by 344 copies. Both miR-10-5p and its respective star sequence had higher numbers of variants than other miRNAs (Figure 2). There were some exceptions to this general picture: miR-214 was represented by 47 copies, while its star sequence miR-214* possessed 6,579; in the case of miR-745, both miR-745 itself and miR-745* had high copy numbers of 2,342 and 5,809. Because miRNAs or their star sequences are likely to be quickly metabolized and removed if they are not required by the cells, the presence of star sequences in such high copy number suggests that these star sequences may fulfill some gene regulatory function(s) in *O. turkestanicum*.

### Phylogenetic Analysis

**Figure 3:**
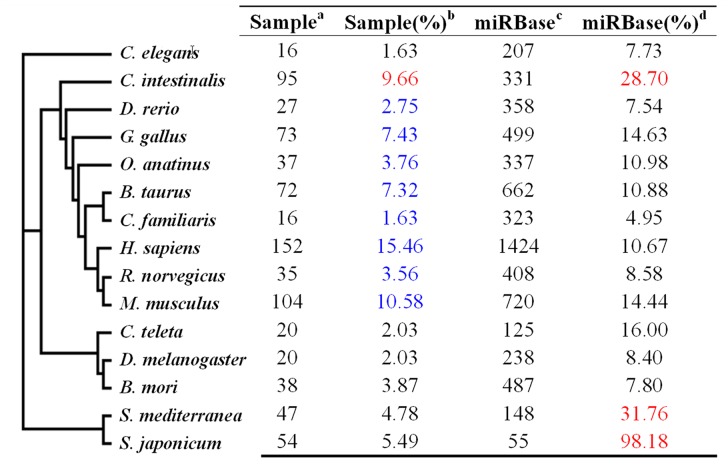
Taxonomic distribution of the miRNA dataset of ***Orientobilhazia turkestanicum*** **.** a means miRNAs of the *O. turkestanicum* that matched with miRNAs of the species listed on the left; b shows the percentage of miRNAs of the *O. turkestanicum* matching to total miRNAs of the 15 species listed (see text for details); c number of miRNAs deposited in the miRBase database; d number of *O. turkestanicum* miRNAs number expressed as a percentage of miRNAs in miRBase, to show the coverage rate of miRNAs in *O. turkestanicum*. Taxonomic classification: nematodes (group I, including *C. elegans*), vertebrates (group II, from *C. intestinalis* to *M. musculus*), insects (group III, from *C. teleta* to *B. mori*), and platyhelminths (group IV, including *S. mediterranea* and *S. japonicum*).

Homologues of the miRNA dataset of *O. turkestanicum* were distributed amongst 55 different taxa, including free living species. Of these, the top 15 species with the most homologs of *O. turkestanicum* miRNAs are shown in Figure 3. The phylogenetic relationship tree on the left of the figure was constructed from NCBI taxonomy website. These 15 species represent 4 groups; nematodes, platyhelminths, insects and vertebrates. Blue color showed percentage of vertebrate miRNAs; Red color showed the highest coverage percentages of miRNAs in the *O. turkestanicum* dataset to miRNAs deposited in the miRBase. The highest coverage percentages of 31.76% and 98.18% were found in platyhelminthes including *S. mediterranea* and *S. japonicum*, followed by coverage percentage in *Ciona intestinalis*. *C. intestinalis* is a protochordate, considered as a transitional species between invertebrate to vertebrate. For the purposes of this analysis, it has been classified as a vertebrate.

Both *Homo sapiens* and *Mus musculus* had high numbers of homologs of *O. turkestanicum* miRNAs, [152 (15.46%) and 104 (10.58%) respectively], followed by *C. intestinalis* with 95 (9.66%). In total, vertebrate species (including *C. intestinalis*) accounted for 62.16% of the *O. turkestanicum* miRNA homologs but platyhelminths, including the closely related species *Schistosoma japonicum*, only accounted for 10.12% of the homologous miRNAs. A further 1.63% of the *O. turkestanicum* miRNA homologs were from the nematode *Caenorhabditis elegans*. These proportions may be influenced by the numbers of miRNAs deposited in the miRBase - the “distilled” miRNA dataset of *O. turkestanicum* used for this analysis was obtained by matching sequencing reads with miRNAs deposited in the miRBase. To address this, we have calculated the number of miRNAs of each species in the database, and then expressed *O. turkestanicum* miRNAs as the percentages of this number. For example, for *C. elegans*, there were 207 miRNAs deposited in the miRBase database, with 16 miRNAs which possessed homology to those of *O. turkestanicum*. Therefore, the percentage coverage for this species was 7.73% (16/207). By this calculation method, *S. japonicum* has a very high percentage of miRNA coverage (98.18%); which means that almost all of the currently-known miRNAs of *S. japonicum* deposited in the miRBase are also present in *O. turkestanicum*. In addition to *S. japonicum*, another platyhelminth, *Schmidtea mediterranea*, possessed 31.76% coverage, followed by *C. intestinalis* with 28.70%. *O. turkestanicum* possessed miRNAs of ancient origin such as those found in nematodes and platyhelminths, prior to the evolution of insects and coelomates.

**Figure 4:**
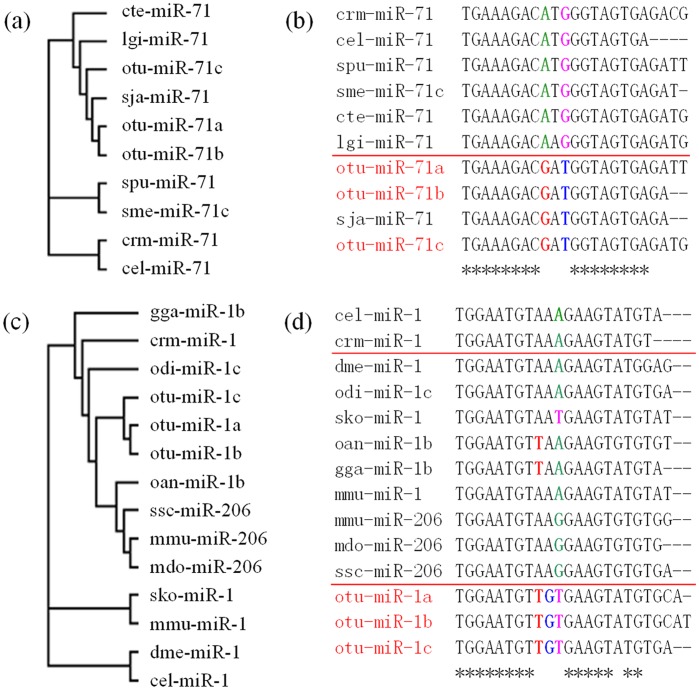
Phylogenetic analyses of miR-1 and miR-71 families. Panels a and b : Phylogenetic analysis of the miR-1 family shows a similar evolution pattern in both platyhelminthes *O. turkestanicum* and *Schistosoma japonicum*. **Panels c and d**: Phylogenetic analysis of the miR-71 family shows distinct evolutionary differences between *O. turkestanicum* miRNA and those from other species. Full name and taxonomic classification: (a) cte: *Capitella teleta*; lgi: *Lottia gigantea*; otu: *Orientobilhazia turkestanicum*; sja: *Schistosoma japonicum*; spu: *Strongylocentrotus purpuratus*; sme: *Schmidtea mediterranea*; crm: *Caenorhabditis remanei*; cel: *Caenorhabditis elegans*; (b) up: nematodes and other invertebrates; down: variants of otu showed same mutation characteristics, but different with nematodes and other invertebrates above; (c) gga: *Gallus gallus*; crm: *Caenorhabditis remanei*; odi: *Oikopleura dioica*; oan: *Ornithorhynchus anatinus*; ssc: *Sus scrofa*; mmu: *Mus musculus*; mdo: *Monodelphis domestica*; sko: *Saccoglossus kowalevskii*; dme: *Drosophila melanogaster*; (d) up: nematodes; middle: Hemichordata, insect, bird, mammals; down: platyhelminthes. Star line (“*”): consensus sequence.

Two miRNAs (miR-1 and miR-71) of *O. turkestanicum* were randomly selected for further phylogenetic analysis. For each of these two miRNAs of *O. turkestanicum*, three homologous were selected and named with the suffix a, b or c. For example, three homologous of miR-1 of *O. turkestanicum* were named as otu-miR-1a, otu-miR-1b, and otu-miR-1c. Other miR-1 and miR-71 family members from other species were also downloaded from the miRBase for the analysis. The phylogenetic relationships of the two miRNAs are shown in Figure 4. The seed regions, “2–8”, of miR-1 and miR-71 were conserved and 12–18 were relatively conserved (Figure 4b, d). Within the miR-71 family, three miRNAs of *O. turkestanicum* grouped with those of *S. japonicum*, and possessed the same nucleotide mutations at the 9 to 11 positions, with a “transversion” from “A” to “G” at the 9th position; “G” to “T” at the 11th position, and a “transformation” at the 10th position from “T” to “A” (Figure 4a, b). Nucleotide sequences of the miRNAs of *O. turkestanicum* showed the same evolutionary characters as *S. japonicum*, which indicated similar evolutionary patterns in these parasites and a characteristic evolutionary direction of *O. turkestanicum* miRNAs similar to that which we have described for the parasite *Clonorchis sinensis* [23]. The miR-1 family included homologous miRNAs from nematodes (cel-miR-1 and crm-miR-1), insects (such as dme-miR-1), and vertebrates (such as mmu-miR-1 and 206), but the otu-miR-1 possessed a transformation in the 9th position from “A” to “T” and a transversion from “A” to “G” at the 10th position (Figure 4c, d). The characteristics of miR-1 of *O. turkestanicum* suggested a common ancient origin with those of *Caenorhabditis elegans* and *Oikopleura dioica*, but with a subsequent rapid and distinct evolutionary pattern. These evolutionary characteristics suggested that *O. turkestanicum* has a similar evolutionary pattern to that of *S. japonicum*, with differences from other, non-parasitic animals.

### Target and Function Prediction

All of the 828 known miRNAs were used for target analysis by searching against the cDNA dataset with annotation of blood flukes, and 518 miRNAs were successfully predicted (Figure S1). Prediction of functions of the best matched targets of each known miRNAs revealed that targets of some miRNAs were related to energy metabolism, including NADH dehydrogenase (miR-4144-3p, miR-1837, miR-125b*, and miR-36a*) and ATPase (miR-3666, miR-4115-5p, miR-4038-3p, miR-3668, miR-3559-3p, and miR-503); some of them were related to transcription initiation factor (miR-369-3p, miR-139, miR-4082-3p and miR-4148-5p), and splicing factor (miR-23a*, miR-767-3p, miR-463, miR-598 and miR-2881). Some miRNAs were related to signal transduction (miR-532-3p and miR-236) and growth factor receptor (miR-1942 and miR-71a). Five miRNAs (miR-4020b-5p, miR-92e-5p, miR-252, miR-4150-5p and miR-4101-3p) showed perfectly complementary to zinc finger protein. Interestingly, 3 miRNAs were found to be related to the egg protein (egg protein CP111, miR-320c, miR-2444) and egg antigen (major egg antigen p40, miR-2807c*); and another 3 miRNAs were related to heat shock proteins (HSP90 with miR-36c; HSP60 with miR-486-5p and HSP10 with miR-669 g). The complementary structure of miRNAs with their star sequences are shown in Figure S2.

**Figure 5:**
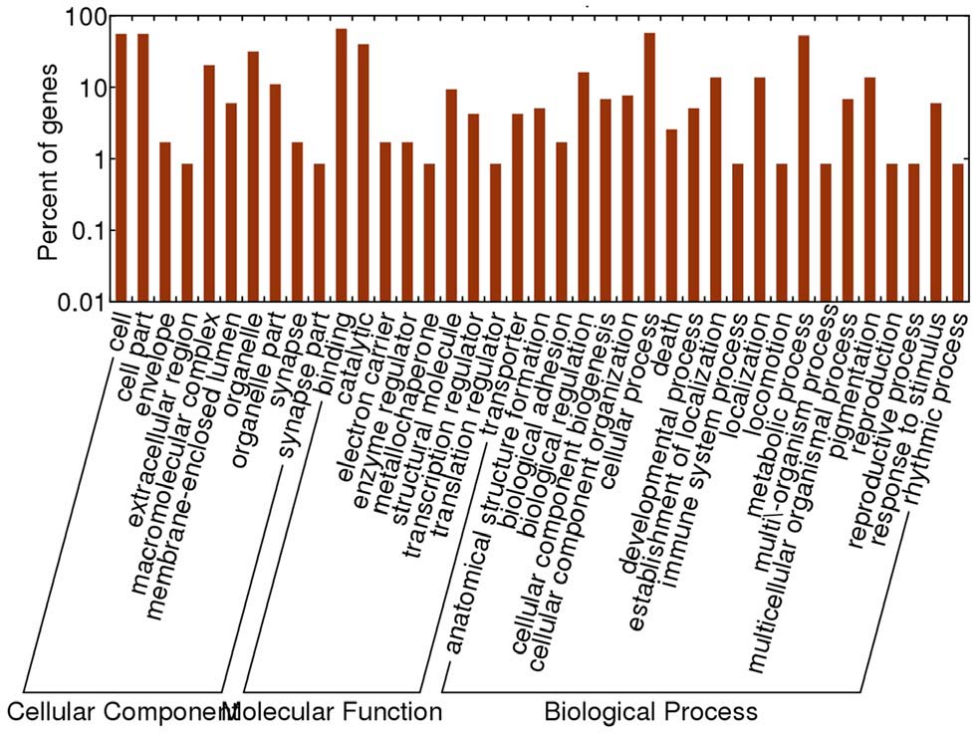
Enrichment analysis to the predicted targets of miRNA of *Orientobilhazia turkestanicum*.

Enriched analysis showed that the predicted functions of the targets were focused on catalytic and binding functions, which were followed by structural molecules, transcription regulators and translation regulators. For biological processes, biological regulation, cellular processes, and metabolic processes accounted for most of the processes (Figure 5).

### Novel miRNA Prediction and Quantification

A total of 979,228 (71.36%) un-annotated, unique reads that matched elements of the *S. japonicum* genome were marked as potential novel miRNA candidates. The secondary structure of the inverted repeat (predicted by Mfold) revealed 7 novel miRNAs. The 7 predicted novel miRNAs mapped to different elements of *S. japonicum* genome, four were located on the positive strand, while 3 were located on the negative strand. With the exception of Otu-miR-02, none of the novel miRNAs had star sequences.

The relative expression levels of five of the novel miRNAs (Otu-miR-01 to Otu-miR-05) in *O. turkestanicum* were quantified using modified stem-loop RT-PCR (Table 2, “expression level”). Otu-miR-01, Otu-miR-02, and Otu-miR-04 had expression levels 4–5 times higher than the endogenous reference miRNA, while Otu-miR-05 had a similar expression level to that of the endogenous reference gene. For Otu-miR-03, no expression level could be determined accurately.

**Table 2:** Detailed information of the seven novel miRNAs identified in *Orientobilharzia turkestanicum*.

niRNA	Location	ΔG (kcal/mol)	Size	Star sequence	Expression level
Otu-miR-01	SJC_S000092∶515421:515509:+89	−0.2	20	N	5.28±0.4
Otu-miR-02	SJC_S000254∶283546:283638:+93	−27.5	22	Y	4.65±0.13
Otu-miR-03	SJC_S000721∶99266:99349:-84	−25.9	20	N	N/A
Otu-miR-04	SJC_S000804∶15542:15636:+95	−19.0	20	N	5.64±0.31
Otu-miR-05	SJC_S002752∶13824:13901:-78	−22.7	20	N	1±0.15
Otu-miR-06	SJC_S007735∶3474:3550:- 77	−30.3	20	N	N/A
Otu-miR-07	SJC_S020269∶1123:1195:+73	−22.0	20	N	N/A

## Discussion

*Orientobilharzia* spp. infect humans and animals in a range of countries. In China, for example, *O. turkestanicum* has a wide geographical distribution, taking in more than 24 provinces [32]. Outbreaks of *Orientobilharzia* spp. usually follow environmental flooding and may result in a high incidence of disease, with associated deaths and economic loss. In the life cycle of *Orientobilharzia* spp., the cercariae are released from intermediate snail hosts (such as *Lymnaea* and *Indoplanorbis* sp.) into still water and humans or animals usually become infected by penetration of the cercariae through the skin during contact with the contaminated water during swimming, working or wading in these environments [1]. As *O. turkestanicum* is a member of the *Schistosoma*, the invasion mechanism and life cycle of this species is similar to that of *S. japonicum* [20]. Therefore, the investigation of lifestyle in *O. turkestanicum* will assist in the development of novel technologies for the control of both *Orientobilharzia* and related schistosomes.

In this study, we obtained 13.78 million raw reads which corresponded to 13.48 million high quality reads, including 828 conserved miRNAs from adult *O. turkestanicum*. Two distinct characteristics of the small RNAs of adult *O. turkestanicum* were that the majority of them were 22 nt in length and that there was a higher percentage of repeat reads than that found in other parasites. For the three peaks of small RNAs observed at 20 nt, 22 nt and at 28 nt, it was found that most of these reads were rRNA and tRNA except 22 nt, of which 42.36% were miRNAs.

Phylogenetic analysis showed that some of the miRNAs of *O. turkestanicum* were distributed in a large range of taxa from nematodes, platyhelminths, insects to vertebrates. On the other hand, as the examples of miR-1 and miR-71 showed, *O. turkestanicum* has a similar evolutionary pattern to that of *S. japonicum*, with differences from other, non-parasitic animals. This phenomenon might indicate an ancient origin but fast evolution of the miRNAs, which has a directed mutation trend. Target and function predicted analysis of the fluke showed that the miRNAs of *O. turkestanicum* had a wide range of targets, and some of the targets were essential to the parasite, such as NADH dehydrogenase and ATPase which belonged to energy metabolism, and signal transduction, etc. This phenomenon was certified by Enrichment analysis, which showed that catalytic, binding functions, transcription regulators and translation regulators were enriched in the present dataset of targets of *O. turkestanicum*.

Another two targets named egg protein and major egg antigen p40 were also found. The major egg antigen p40 in *S. mansoni* was reported as a leading anti-pathology schistosomal vaccine candidate [33]. *S. mansoni* infections cause granulomatous response of host tissue by deposited eggs which subsequently result in tissue fibrosis. It was found that the major egg antigens have noticed desensitizing role to the CD4^+^ Th cells that mediate granuloma formation, and therefore ameliorate the clinical signs of schistosomiasis. Purified egg antigen resulted in reduced deposition, decreased fibrosis, and granuloma formation inhibition in *S. mansoni* [33]. In *Paragonimus westermani*, a recombinant protein of egg was also produced and tested as an antigen, which was found to be distributed only in eggs and uteri of adult worms of *P. westermani* [34]. Therefore, the egg target revealed here indicated a potential role of miRNAs to modulate expression of egg antigens and the immune response against schistosomal infections.

Furthermore, the predicted amino acid sequence of the egg antigen showed 35–40% of identity to heat shock proteins (HSP) from *S*. *japonicum*, *S*. *mansoni*, and *Taenia saginata* [34], which indicated a closely relationship of egg antigen and HSP. It was reported that Hsp90 is an evolutionarily conserved molecular chaperone, and it is essential in all eukaryotes studied for 3 aspects: 1) it is a part of the steroid hormone receptor complex; 2) it is a chaperone that helps proteins to fold; 3) its activity in response to specific environmental stimuli [35]–[37]. Hsp60 refolds nuclear-encoded proteins after passage through organellar membranes, and the Hsp60 RNA and protein expression can be strongly induced by high incubating temperature (42 degrees C) [38]. The nucleotide and protein sequences for HSP70 were found to be 98% and 99% identical between different liver fluke species, such as *Fasciola hepatica* and *F. gigantica*, with expression to be higher in *F. gigantica* than in *F. hepatica*, which might represent a biochemical marker of the stress response of *F. gigantica* [39]. Therefore, miRNAs with the egg antigen and HSP as targets may have prospect as leads in anti-pathology strategies against trematodes.

In conclusion, the present study investigated the miRNA expression profile of adult *O. turkestanicum*. This study is the first large-scale characterization of *O. turkestanicum* miRNAs, which provides novel resources for better understanding the complex biology of this zoonotic parasite, and has implications for the effective control of the disease it causes.

## Supporting Information

Figure S1 **Targets predicted with the cDNA dataset of the blood fluke.** The targets were analyzed with RNAhybrid under under default parameters. Two extra parameters were performed to the result: 1) the △△G was set as lower than -25 kcal/mol; 2) P-value was set as ≧ 0.05.
(PDF)

Figure S2 **Complementary Structure of miRNA with their star sequences.**
(PDF)
