# Supplementary material for: Characterization of MicroRNAs from Orientobilharzia turkestanicum, a Neglected Blood Fluke of Human and Animal Health Significance
Source: PLoS One. 2012 Oct 10;7(10):e47001. doi: 10.1371/journal.pone.0047001 (PMC3468544; doi:10.1371/journal.pone.0047001)
Supplement: Figure S1 — Targets predicted with the cDNA dataset of the blood fluke. The targets were analyzed with RNAhybrid under under default parameters. Two extra parameters were performed to the result: 1) the △△G was set as lower than -25 kcal/mol; 2) P-value was set as ≧ 0.05. (PDF) [file pone.0047001.s001.pdf]

## Additional file 1. Targets analysis of all miRNAs of *Orientobilharzia turkestanicum*

| Name | Length | Mfe (kcal/mol) | Number of targets | Name of Best matched Target | KEGG Orthology |
|---|---|---|---|---|---|
| **Novel miRNAs** | | | | | |
| Otu-miR-01 | 20 | -30.2 | 66 | Lipopolysaccharide-induced tumor necrosis factor-alpha factor homolog | / |
| Otu-miR-02 | 22 | -37.3 | 57 | hypothetical protein | / |
| Otu-miR-03 | 20 | -30 | 2 | / | / |
| Otu-miR-04 | 20 | -42.1 | 21 | Conserved hypothetical protein | / |
| Otu-miR-05 | 20 | -29.4 | 35 | Heterogeneous nuclear ribonucleoprotein A2 homolog 1 | / |
| Otu-miR-06 | 20 | -27.2 | 59 | hypotheticial protein | / |
| Otu-miR-07 | 20 | -26.9 | 1 | hypotheticial protein | / |
| Known miRNAs | | | | | |
| miR-1* | 22 | -29.3 | 1270 | / | / |
| miR-23* | 21 | -36.6 | 905 | / | / |
| miR-1721 | 19 | -34.4 | 673 | Transmembrane protein 45B | / |
| miR-2303 | 21 | -31.5 | 637 | / | / |
| miR-2885 | 22 | -40.3 | 354 | U1 small nuclear ribonucleoprotein A | / |
| miR-595 | 20 | -28.9 | 239 | hypothetical protein | / |
| miR-361* | 25 | -27.9 | 202 | H+-transporting ATPase | / |
| miR-3615 | 19 | -36.5 | 190 | / | / |
| miR-3201 | 20 | -32 | 187 | / | / |
| miR-2881 | 20 | -43.3 | 175 | Splicing factor 3A subunit 2 | / |
| miR-1591 | 25 | -40.2 | 171 | / | / |

| | | | | | |
|---|---|---|---|---|---|
| miR-2305 | 18 | -37.4 | 162 | cleavage stimulation factor, 3' pre-RNA, subunit 2, 64kDa | / |
| miR-2861 | 21 | -39.7 | 143 | Ubiquinol-cytochrome-c reductase complex core protein 2, mitochondrial precursor | [EC:1.10.2.2] |
| miR-720 | 19 | -36.6 | 132 | RNA-binding protein 4 | / |
| miR-1260 | 20 | -36.9 | 124 | Krt9 | ko:K07604 type I keratin, acidic |
| miR-1400 | 19 | -35.7 | 121 | Krt9 | ko:K07604 type I keratin, acidic |
| miR-4332 | 21 | -38.4 | 108 | Centrin-3 | / |
| miR-1584 | 21 | -36.4 | 107 | Lysosomal protective protein precursor | [EC 3.4.16.5] |
| miR-769 | 20 | -35.6 | 105 | / | / |
| miR-1587 | 20 | -36.4 | 100 | IPP-2 | / |
| miR-1934* | 18 | -36.3 | 97 | hypothetical protein | / |
| miR-504* | 21 | -40.8 | 94 | putative CCDC28B protein 65.9 | / |
| miR-3058* | 23 | -46 | 91 | hnRNP-K | / |
| miR-431 | 20 | -32.1 | 90 | / | / |
| miR-4300 | 19 | -34 | 88 | / | / |
| miR-669c* | 25 | -38.4 | 86 | ribosomal protein S8 | ko:K02993 small subunit ribosomal protein S7e |
| miR-242 | 22 | -37.2 | 82 | / | / |
| miR-347 | 19 | -39.4 | 78 | / | / |
| miR-194 | 22 | -32.7 | 72 | LSM1 homolog, U6 small nuclear RNA associated | / |
| miR-647 | 25 | -35.9 | 70 | Heterogeneous nuclear ribonucleoprotein K | / |
| miR-4020b-5p | 18 | -33.6 | 70 | Zinc finger protein 207 | / |
| miR-1254 | 21 | -34.1 | 70 | Ahcy | [EC:3.3.1.1] ko:K01251 adenosylhomocysteinase |
| miR-669c | 20 | -32.5 | 69 | / | / |

| | | | | | |
|---|---|---|---|---|---|
| miR-574-5p | 25 | -41.7 | 69 | hypothetical protein | / |
| miR-4320 | 20 | -29.1 | 69 | / | / |
| miR-1346 | 20 | -37.2 | 68 | hypotheticial protein | / |
| miR-92e-5p | 20 | -36.2 | 67 | Zinc finger protein 207 | / |
| miR-423* | 24 | -37.1 | 66 | hypothetical protein | / |
| miR-1607 | 18 | -34.2 | 66 | Ibp2(Intersectin-EH-binding protein 2) | / |
| miR-4281 | 20 | -39.1 | 63 | / | / |
| miR-671-5p | 24 | -38.1 | 61 | Ras GTPase-activating protein-binding protein 1 | [EC:3.6.1.-] |
| miR-1307 | 23 | -36 | 61 | ADP-ribosylation factor 1 GTPase-activating protein | / |
| miR-745* | 23 | -36.8 | 59 | / | / |
| miR-92d-5p | 20 | -31.1 | 57 | hypothetical protein | / |
| miR-1959 | 19 | -35.7 | 57 | / | / |
| miR-4170-5p | 18 | -33.2 | 56 | putative 6-phosphogluconate dehydrogenase | [EC:1.1.1.44] |
| miR-1612 | 24 | -36.6 | 56 | / | / |
| miR-32* | 24 | -30.8 | 55 | / | / |
| miR-1345 | 18 | -32.5 | 55 | putative KH-type splicing regulatory protein | / |
| miR-485-5p | 24 | -33.3 | 53 | Histone H2A | / |
| miR-78 | 21 | -31.9 | 51 | hypothetical protein | / |
| miR-705 | 20 | -36.5 | 51 | CDC37 | ko:K09554 cell division cycle protein 37 |
| miR-4088-5p | 21 | -34 | 48 | / | / |
| miR-125-2* | 19 | -36 | 47 | Tubulin beta-2C chain | / |
| miR-194a | 21 | -32.3 | 46 | / | / |
| miR-92b* | 24 | -34.7 | 45 | lethal (2) 06225 | [EC:3.6.3.14] ko:K02140 F-type H+-transporting ATPase g chain |
| miR-3652 | 20 | -33.6 | 45 | hypothetical protein | / |
| miR-277c* | 21 | -30.9 | 45 | GTPase-activating protein ZNF289 | / |

| | | | | | |
|---|---|---|---|---|---|
| miR-277c | 22 | -30.9 | 44 | Ribosomal protein S11 | ko:K02949 small subunit ribosomal protein S11e |
| miR-99b | 22 | -31.8 | 43 | Ribosomal Protein, Large subunit | ko:K02894 large subunit ribosomal protein L23e |
| miR-27a* | 22 | -34.6 | 42 | / | / |
| miR-148a* | 20 | -30.7 | 42 | Extracellular superoxide dismutase [Cu-Zn] precursor | [EC:1.15.1.1] |
| miR-1231 | 18 | -32.7 | 42 | / | / |
| let-7b | 22 | -31.3 | 42 | hypothetical protein | / |
| miR-2386 | 22 | -30.1 | 41 | / | / |
| miR-129-5p | 21 | -33.6 | 41 | inositol polyphosphate-5-phosphatase E | [EC:3.1.3.36] ko:K01099 phosphatidylinositol-bisphosphatase |
| miR-106b* | 21 | -31.7 | 41 | Small nuclear ribonucleoprotein SM D3 | / |
| miR-980 | 21 | -33.2 | 40 | chloride intracellular channel 4 | ko:K05024 chloride intracellular channel 4 |
| miR-745b | 22 | -33.2 | 40 | chloride intracellular channel 4 | ko:K05024 chloride intracellular channel 4 |
| miR-252 | 19 | -36.8 | 40 | Zinc finger protein 207 | / |
| miR-1649* | 18 | -35 | 40 | / | / |
| miR-3666 | 25 | -35.3 | 39 | ATPase, H+ transporting, lysosomal, V0 subunit c | [EC:3.6.3.14] ko:K02155 V-type H+-transporting ATPase 16kDa proteolipid subunit |
| miR-276a | 24 | -31.4 | 39 | rpl-23 | ko:K02894 large subunit ribosomal protein L23e |
| miR-3622b-5p | 18 | -31.9 | 38 | / | / |
| miR-1937a | 19 | -35.2 | 38 | Heterogeneous nuclear ribonucleoprotein U-like protein 1 | / |
| miR-423-5p | 19 | -30.4 | 37 | putative transformer-2 protein | / |
| miR-1759 | 25 | -37.4 | 36 | hypothetical protein | / |

| | | | | | |
|---|---|---|---|---|---|
| miR-1183 | 24 | -36.5 | 36 | RpL9 | ko:K02940 large subunit ribosomal protein L9e |
| miR-23a* | 22 | -31.4 | 35 | Splicing factor, arginine/serine-rich 7 | / |
| miR-10b | 22 | -32.6 | 35 | Cellular retinaldehyde-binding protein | / |
| miR-205* | 22 | -32.9 | 34 | NAP-1-related protein | / |
| miR-10a-5p | 24 | -34.7 | 34 | Cellular retinaldehyde-binding protein | / |
| miR-10-5p | 22 | -32.8 | 34 | hypotheticial protein | / |
| miR-683 | 20 | -32.6 | 33 | Chromobox protein homolog 1 | / |
| miR-669g | 23 | -32.1 | 33 | heat shock 10kD protein 1 | / |
| miR-466i-5p | 20 | -29.9 | 33 | Basic helix-loop-helix dimerisation region bHLH,domain-containing protein | / |
| miR-2478 | 21 | -33.8 | 33 | cellular nucleic acid-binding protein | / |
| miR-200b* | 22 | -33.9 | 33 | putative choline phosphotransferase 1 | ko:K00994 diacylglycerol cholinephosphotransferase |
| miR-1816 | 21 | -32.2 | 32 | Pre-mRNA-splicing factor cwc15 | / |
| miR-1638 | 21 | -28.9 | 32 | ribosomal protein S14 | ko:K02955 small subunit ribosomal protein S14e |
| miR-339 | 22 | -34 | 30 | RAB family | ko:K07976 Rab family, other |
| miR-3180-3p | 24 | -39.1 | 30 | / | / |
| miR-271 | 22 | -36.7 | 30 | Mastin precursor | [EC:3.4.21.-] |
| miR-483-3p | 18 | -30 | 29 | / | / |
| miR-25 | 22 | -30.2 | 29 | hypothetical protein | / |
| miR-2411 | 20 | -32.6 | 29 | / | / |
| miR-222b | 21 | -32 | 29 | hypothetical protein | / |
| miR-193a-5p | 21 | -32.2 | 29 | hypotheticial protein | / |
| miR-92a | 22 | -30.7 | 28 | putative ATP synthase | [EC:3.6.3.14] ko:K02128 F-type |

| | | | | | |
|---|---|---|---|---|---|
| | | | | | H+-transporting ATPase subunit c |
| miR-2476 | 18 | -31.5 | 28 | hypothetical protein | / |
| miR-277b | 23 | -30.1 | 27 | CRISP-9 | / |
| miR-2489 | 23 | -31.2 | 27 | polyubiquitin | ko:K04551 ubiquitin B |
| miR-4148-5p | 20 | -33.6 | 26 | TATA box binding protein like 2 | ko:K03120 transcription initiation factor TFIID TATA-box-binding protein |
| miR-2b-3p | 23 | -37.8 | 26 | Fh1 | [EC:4.2.1.2] ko:K01679 fumarate hydratase |
| miR-190a-5p | 30 | -32.8 | 26 | ribosomal protein S16 | ko:K02960 small subunit ribosomal protein S16e |
| miR-190 | 24 | -35.6 | 25 | / | / |
| miR-149 | 22 | -32.9 | 25 | hypotheticial protein | / |
| miR-652 | 21 | -34.5 | 24 | may be involved in transcription elongation by mediating interactions between RNA polymerase II and chromatin | / |
| miR-29a | 22 | -33.6 | 24 | large subunit ribosomal protein L10e | / |
| miR-2387 | 25 | -37.3 | 24 | / | / |
| miR-739 | 20 | -36.4 | 23 | hypothetical protein | ko:K04645 clathrin, light polypeptide B |
| miR-604 | 18 | -32 | 23 | KH domain-containing, RNA-binding, signal transduction-associated protein 1 | / |
| miR-3126-5p | 24 | -34.6 | 23 | U3 small nucleolar RNA-interacting protein 2 | / |
| miR-277 | 22 | -28.7 | 23 | putative U5 snRNP-specific protein | / |
| miR-466i-3p | 24 | -46.6 | 22 | hypothetical protein | / |
| miR-3488 | 20 | -33.4 | 22 | hypothetical protein | / |
| miR-1556 | 25 | -35 | 22 | hypotheticial protein | / |
| miR-1386 | 21 | -42.9 | 22 | / | / |
| miR-8-3p | 22 | -28.5 | 21 | small subunit ribosomal protein S30e | / |

| | | | | | |
|---|---|---|---|---|---|
| miR-3095-3p | 24 | -36.4 | 21 | / | / |
| miR-24 | 22 | -33.5 | 21 | High mobility group protein 20A | / |
| miR-184b | 20 | -34.1 | 21 | / | / |
| miR-1786 | 21 | -32.9 | 21 | Import inner membrane translocase subunit TIM44, mitochondrial precursor | / |
| miR-147 | 21 | -32.8 | 21 | Cathepsin B-like cysteine proteinase precursor | [EC:3.4.22.-] |
| miR-99a | 21 | -32.3 | 20 | / | / |
| miR-3176 | 19 | -31.9 | 20 | / | / |
| miR-3118 | 24 | -34 | 20 | putative 40S ribosomal protein S9 | ko:K02997 small subunit ribosomal protein S9e |
| miR-2397 | 20 | -34.4 | 20 | PSMC3 | ko:K03065 26S proteasome regulatory subunit T5 |
| miR-1274b | 20 | -37.4 | 19 | / | / |
| miR-3482-3p | 23 | -44.6 | 18 | hypothetical protein | / |
| miR-320a | 22 | -34.3 | 18 | Acidic leucine-rich nuclear phosphoprotein 32-related protein | / |
| miR-236 | 24 | -31.7 | 18 | signal peptidase complex subunit 2 homolog | / |
| miR-2230* | 23 | -34.5 | 18 | Peptidase M8, leishmanolysin | / |
| miR-2200* | 21 | -32.7 | 18 | hypothetical protein | / |
| miR-1603 | 18 | -31.9 | 18 | / | / |
| miR-765 | 21 | -33.6 | 17 | / | / |
| miR-749 | 25 | -40.4 | 17 | Basic-leucine zipper (bZIP) transcription factor | / |
| miR-677 | 21 | -36.7 | 17 | High mobility group protein DSP1 | / |
| miR-423-3p | 23 | -37.6 | 17 | / | / |
| miR-3483-5p | 22 | -40.1 | 17 | hypothetical protein | / |
| miR-339b | 20 | -33.9 | 17 | Centaurin-alpha 1 (Putative MAPK-activating protein | / |

| | | | | | |
|---|---|---|---|---|---|
| | | | | PM25) | |
| miR-2440 | 19 | -31.8 | 17 | / | / |
| miR-150* | 20 | -34 | 17 | Ubiquinol-cytochrome-c reductase complex core protein 2, mitochondrial precursor | [EC:1.10.2.2] |
| miR-767-3p | 24 | -33.4 | 16 | Splicing factor, arginine/serine-rich 7 | / |
| miR-2993 | 20 | -33.7 | 16 | / | / |
| miR-28-3p | 22 | -34.7 | 16 | Heterogeneous nuclear ribonucleoprotein K | / |
| miR-2785 | 21 | -31.5 | 16 | / | / |
| miR-181a-2* | 24 | -37.5 | 16 | / | / |
| miR-1614* | 19 | -31.7 | 16 | / | / |
| miR-1 | 21 | -33 | 16 | / | / |
| miR-87b | 22 | -30.9 | 15 | putative ribosomal protein L31 | ko:K02910 large subunit ribosomal protein L31e |
| miR-87 | 22 | -29 | 15 | putative ribosomal protein L31 | ko:K02910 large subunit ribosomal protein L31e |
| miR-500a* | 23 | -33.8 | 15 | ARMET-like protein precursor | / |
| miR-4131-3p | 20 | -33.5 | 15 | hypotheticial protein | / |
| miR-4106-3p | 21 | -26.1 | 15 | hypotheticial protein | / |
| miR-3492 | 24 | -32.8 | 15 | / | / |
| miR-2065 | 19 | -30 | 15 | glutathione S-transferase M1 | [EC:2.5.1.18] ko:K00799 glutathione S-transferase |
| miR-2020 | 25 | -34.9 | 15 | / | / |
| miR-1794 | 24 | -31.8 | 15 | / | / |
| miR-84 | 23 | -32.1 | 14 | ubiquitin coniugating enzyme 3b | [EC:6.3.2.19] ko:K02207 ubiquitin-conjugating enzyme E2 |
| miR-745 | 22 | -33.2 | 14 | / | / |

| | | | | | |
|---|---|---|---|---|---|
| miR-341 | 19 | -34.2 | 14 | / | / |
| miR-2537* | 20 | -35.5 | 14 | cytosolic malate dehydrogenase | [EC:1.1.1.37] ko:K00026 malate dehydrogenase |
| miR-1701 | 20 | -31.7 | 14 | / | / |
| miR-153* | 20 | -33 | 14 | hypothetical protein | / |
| miR-133 | 23 | -33.8 | 14 | Heterogeneous nuclear ribonucleoprotein K | / |
| miR-92b | 22 | -34.7 | 13 | THO complex subunit 4 | / |
| miR-532-3p | 22 | -35.9 | 13 | signal transduction-associated protein 1 | / |
| miR-463 | 20 | -30.5 | 13 | Splicing factor, arginine/serine-rich 7 | / |
| miR-4115-5p | 22 | -31 | 13 | ATPase, H+ transporting, lysosomal 31kDa, V1 subunit E | [EC:3.6.3.14] ko:K02150 V-type H+-transporting ATPase subunit E |
| miR-3475 | 21 | -30.3 | 13 | putative ubiquitin-conjugating enzyme | / |
| miR-320c | 19 | -31.1 | 13 | Egg protein CP111 | / |
| miR-2450b | 24 | -32.1 | 13 | GrpE-like protein | ko:K03687 molecular chaperone GrpE |
| miR-1837 | 25 | -35.4 | 13 | NADH dehydrogenase (ubiquinone) 1 beta subcomplex 9 | [EC:1.6.5.3 1.6.99.3] |
| miR-1639 | 18 | -31.3 | 13 | / | / |
| miR-146b-3p | 23 | -33.9 | 13 | Voltage-dependent anion-selective channel protein 2 | / |
| miR-106b | 21 | -33.3 | 13 | cyclin B1 interacting protein 1 | [EC:6.3.2.-] |
| miR-1010* | 21 | -30.4 | 13 | Dynein light chain 2, cytoplasmic | / |
| miR-7c | 22 | -30.9 | 12 | / | / |
| miR-4255 | 20 | -33 | 12 | / | / |
| miR-4139-3p | 22 | -31.1 | 12 | TAR DNA-binding protein 43 | / |
| miR-375-3p | 20 | -32.6 | 12 | Brix domain-containing protein 1 | / |
| miR-3500 | 19 | -32.3 | 12 | hypothetical protein | / |
| miR-253 | 21 | -32.2 | 12 | conserved hypothetical protein | / |

| | | | | | |
|---|---|---|---|---|---|
| miR-1939 | 22 | -33.2 | 12 | / | / |
| miR-107 | 23 | -38.8 | 12 | putative Vesicle-associated membrane protein-associated protein A | / |
| miR-103 | 23 | -36.5 | 12 | putative Vesicle-associated membrane protein-associated protein A | / |
| miR-669e | 21 | -31.4 | 11 | hypothetical protein | / |
| miR-520d-5p | 18 | -31.7 | 11 | putative ATP-dependent RNA helicase DDX17 | [EC:3.6.1.-] |
| miR-4035-3p | 21 | -33.7 | 11 | hypothetical protein | / |
| miR-3923 | 25 | -33.7 | 11 | / | / |
| miR-361-5p | 22 | -32.7 | 11 | / | / |
| miR-3432 | 24 | -34.1 | 11 | / | / |
| miR-320 | 21 | -28.9 | 11 | putative ATP synthase | [EC:3.6.3.14] ko:K02128 F-type H+-transporting ATPase subunit c |
| miR-31b | 23 | -34.3 | 11 | / | / |
| miR-3021 | 24 | -35.1 | 11 | Flot1 | ko:K07192 flotillin |
| miR-295 | 23 | -29.1 | 11 | / | / |
| miR-2834 | 20 | -33.4 | 11 | calreticulin | ko:K08057 calreticulin |
| miR-193b* | 21 | -34.5 | 11 | / | / |
| miR-181b | 21 | -35 | 11 | / | / |
| miR-1269 | 26 | -34.8 | 11 | / | / |
| miR-124-5p | 22 | -32.2 | 11 | / | / |
| miR-105 | 24 | -28.7 | 11 | small subunit ribosomal protein S27e | / |
| miR-541 | 20 | -33.6 | 10 | NECAP-like protein CG9132 | / |
| miR-518d-5p | 23 | -31 | 10 | / | / |
| miR-466f-3p | 21 | -33.1 | 10 | / | / |
| miR-4294 | 20 | -34.6 | 10 | / | / |

| | | | | | |
|---|---|---|---|---|---|
| miR-34 | 20 | -28.4 | 10 | Huntingtin-interacting protein HYPK | / |
| miR-3229 | 22 | -30 | 10 | Nuclear ribonuclease Z | [EC:3.1.26.11] |
| miR-1642 | 26 | -35.3 | 10 | Glioma pathogenesis-related protein 1 precursor | / |
| miR-151-3p | 22 | -35.7 | 10 | / | / |
| miR-1290 | 20 | -31.3 | 10 | / | / |
| bantam | 22 | -27.4 | 10 | hypothetical protein | / |
| miR-486-3p | 19 | -29.1 | 9 | hypothetical protein | / |
| miR-449d | 18 | -30.5 | 9 | hypothetical protein | / |
| miR-3479-5p | 23 | -29.6 | 9 | hypotheticial protein | / |
| miR-30e | 23 | -32.8 | 9 | / | / |
| miR-2941-1* | 20 | -34.2 | 9 | succinate dehydrogenase complex, subunit B, iron sulfur | [EC:1.3.5.1] ko:K00235 succinate dehydrogenase (ubiquinone) iron-sulfur protein precursor |
| miR-1616 | 21 | -34.1 | 9 | hypothetical protein | / |
| miR-148b-3p | 22 | -28.9 | 9 | / | / |
| miR-138 | 18 | -30.8 | 9 | Histone H2B | / |
| miR-96a | 23 | -32.8 | 8 | / | / |
| miR-960 | 22 | -29.2 | 8 | / | / |
| miR-555 | 19 | -31.3 | 8 | Ras-related protein Rab-4A | / |
| miR-3505 | 25 | -35.6 | 8 | glyoxalase I | [EC:4.4.1.5] ko:K01759 lactoylglutathione lyase |
| miR-320e | 19 | -28.9 | 8 | putative Brain protein 44-like | / |
| miR-31 | 22 | -33.2 | 8 | Peroxiredoxin 5037 | [EC:1.11.1.15] ko:K03386 peroxiredoxin (alkyl hydroperoxide reductase subunit C) |
| miR-30e-5p | 24 | -32.8 | 8 | / | / |
| miR-278 | 21 | -34.6 | 8 | / | / |

| | | | | | |
|---|---|---|---|---|---|
| miR-203 | 21 | -30.8 | 8 | Dfh | / |
| miR-1992-3p | 18 | -32.4 | 8 | Rhabdoid tumor deletion region protein 1 | / |
| miR-100 | 21 | -27.4 | 8 | hypotheticial protein | / |
| miR-71a | 25 | -35 | 7 | growth factor receptor bound protein 2 | ko:K04364 growth factor receptor-binding protein 2 |
| miR-532-5p | 22 | -33.2 | 7 | DnaJ (Hsp40) homolog, subfamily A, member 1 | ko:K09502 DnaJ homolog, subfamily A, member 1 |
| miR-429 | 22 | -31.9 | 7 | putative Proline synthetase co-transcribed bacterial homolog protein | / |
| miR-4115-3p | 19 | -30.9 | 7 | Sdhb | [EC:1.3.5.1] ko:K00235 succinate dehydrogenase (ubiquinone) iron-sulfur protein precursor |
| miR-4038-3p | 19 | -27.9 | 7 | ATPase, F0 complex, subunit E, mitochondrial,domain-containing protein | / |
| miR-375 | 22 | -34.4 | 7 | Brix domain-containing protein 1 | / |
| miR-36-3p | 23 | -35.1 | 7 | Immunogenic miracidial antigen 8I' | / |
| miR-330-3p | 21 | -37.9 | 7 | putative dihydrolipoamide S-acetyltransferase (E2 component of pyruvate dehydrogenase complex) | ko:K00627 pyruvate dehydrogenase E2 component |
| miR-2d-5p | 21 | -34.6 | 7 | / | / |
| miR-277d-3p | 24 | -28.5 | 7 | rps-21 | ko:K02971 small subunit ribosomal protein S21e |
| miR-2549 | 18 | -35.2 | 7 | Heterogeneous nuclear ribonucleoprotein A2 homolog 1 | / |
| miR-2231 | 18 | -30.9 | 7 | / | / |
| miR-2205 | 22 | -34.2 | 7 | / | / |
| miR-196d | 22 | -30.8 | 7 | / | / |
| miR-1942 | 20 | -31.7 | 7 | Growth factor receptor-bound protein 2 | / |

| | | | | | |
|---|---|---|---|---|---|
| miR-1274a | 22 | -34.3 | 7 | / | / |
| miR-125b* | 21 | -33.2 | 7 | NADH dehydrogenase (ubiquinone) 1 alpha subcomplex 8 | [EC:1.6.5.3 1.6.99.3] |
| miR-124-3p | 21 | -33.5 | 7 | rps-26 | ko:K02976 small subunit ribosomal protein S26e |
| miR-598 | 20 | -30 | 6 | Splicing factor, arginine/serine-rich 7 | / |
| miR-509a | 24 | -30.8 | 6 | isoamyl acetate-hydrolyzing esterase 1 homolog | / |
| miR-506 | 19 | -29.1 | 6 | succinate dehydrogenase complex | [EC:1.3.5.1] ko:K00236 succinate dehydrogenase (ubiquinone) cytochrome b subunit precursor |
| miR-466b-1* | 22 | -31.6 | 6 | hypothetical protein | / |
| miR-4150-5p | 19 | -35.7 | 6 | Zinc finger protein | / |
| miR-4101-3p | 19 | -28.5 | 6 | Zinc finger CDGSH domain-containing protein 1 | / |
| miR-377* | 24 | -32.8 | 6 | Aquaporin-9 (AQP-9) (Small solute channel 1) | / |
| miR-302b | 18 | -31.5 | 6 | hypotheticial protein | / |
| miR-2c-5p | 22 | -30.6 | 6 | / | / |
| miR-288 | 22 | -30.8 | 6 | hypothetical protein | / |
| miR-2269 | 19 | -29.4 | 6 | / | / |
| miR-196a | 22 | -32.9 | 6 | / | / |
| miR-1967 | 24 | -33.8 | 6 | U3 small nucleolar RNA-interacting protein 2 | / |
| miR-196 | 21 | -31.6 | 6 | / | / |
| miR-184 | 22 | -32 | 6 | / | / |
| miR-1480 | 24 | -40.2 | 6 | GPI-anchored membrane protein 1 | / |
| let-7 | 21 | -32.9 | 6 | DNMAP1 | / |
| miR-509-3p | 21 | -32.9 | 5 | LIM/homeobox protein Lhx5 | / |
| miR-483 | 20 | -31.5 | 5 | NAP-1-related protein | / |

| | | | | | |
|---|---|---|---|---|---|
| miR-4289 | 19 | -29.8 | 5 | / | / |
| miR-4144-3p | 18 | -30.2 | 5 | NADH dehydrogenase (ubiquinone) Fe-S protein 8 | [EC:1.6.5.3 1.6.99.3] ko:K03941 NADH dehydrogenase (ubiquinone) Fe-S protein 8 |
| miR-3692* | 23 | -35.2 | 5 | hypotheticial protein | / |
| miR-3535 | 26 | -41.2 | 5 | ATP-dependent rRNA helicase RRP3 | [EC:3.6.1.-] |
| miR-330 | 20 | -31.3 | 5 | hypothetical protein | ko:K07567 TdcF protein |
| miR-31b-1* | 21 | -31.8 | 5 | putative UDP-galactose-4-epimerase | ko:K01784 UDP-glucose 4-epimerase |
| miR-2997 | 21 | -29.7 | 5 | prefoldin subunit 4 | ko:K09550 prefoldin subunit 4 |
| miR-2955 | 21 | -34.2 | 5 | / | / |
| miR-281 | 22 | -30.1 | 5 | / | / |
| miR-2772a | 19 | -30.5 | 5 | / | / |
| miR-2448 | 24 | -36.3 | 5 | / | / |
| miR-23b | 23 | -31.6 | 5 | / | / |
| miR-224 | 20 | -33.7 | 5 | putative alpha-1,3-mannosyltransferase | / |
| miR-2136 | 20 | -32.1 | 5 | SNAP-alpha | / |
| miR-205 | 23 | -33.9 | 5 | / | / |
| miR-2032a | 18 | -30.4 | 5 | / | / |
| miR-1682 | 20 | -34.4 | 5 | SAPK substrate protein 1 | / |
| miR-16 | 22 | -31 | 5 | / | / |
| miR-1381 | 19 | -30.4 | 5 | / | / |
| miR-1376 | 19 | -32.2 | 5 | hypothetical protein | / |
| miR-1247* | 18 | -34.8 | 5 | / | / |
| miR-107b | 24 | -32 | 5 | hypothetical protein | / |
| miR-106a | 22 | -32.7 | 5 | / | / |
| miR-76 | 22 | -29.1 | 4 | / | / |
| miR-75* | 20 | -39.6 | 4 | / | / |

| | | | | | |
|---|---|---|---|---|---|
| miR-632 | 19 | -32.1 | 4 | Phosducin-like protein 3 | / |
| miR-486-5p | 21 | -31.8 | 4 | Heat shock protein 60 | ko:K04077 chaperonin GroEL |
| miR-4130-3p | 20 | -29.4 | 4 | / | / |
| miR-4121-3p | 19 | -31.9 | 4 | hypothetical protein | / |
| miR-4052-3p | 21 | -33.8 | 4 | / | / |
| miR-378b | 21 | -34.2 | 4 | VTI1A | ko:K08493 vesicle transport through interaction with t-SNAREs 1 |
| miR-36a* | 20 | -30.2 | 4 | isocitrate dehydrogenase (NAD+) | [EC:1.1.1.41] |
| miR-30d* | 24 | -31.6 | 4 | Histidine-rich glycoprotein precursor | / |
| miR-302c | 22 | -32 | 4 | / | / |
| miR-2779 | 22 | -32.1 | 4 | hypothetical protein | / |
| miR-272 | 20 | -26.2 | 4 | / | / |
| miR-2261 | 20 | -28.2 | 4 | / | / |
| miR-221 | 23 | -29.2 | 4 | hypotheticial protein | / |
| miR-214 | 18 | -31.6 | 4 | Transmembrane emp24 domain-containing protein 10 precursor | / |
| miR-210 | 18 | -30.1 | 4 | / | / |
| miR-17-5p | 26 | -34.3 | 4 | Transmembrane protein 112 | / |
| miR-16b | 22 | -31.9 | 4 | / | / |
| miR-1541 | 19 | -31.8 | 4 | coenzyme Q2 homolog, prenyltransferase | [EC:2.5.1.-] ko:K06125 4-hydroxybenzoate hexaprenyltransferase |
| miR-142-3p | 21 | -30.5 | 4 | / | / |
| miR-139 | 19 | -32.6 | 4 | eIF-4E protein | ko:K03259 translation initiation factor eIF-4E |
| miR-1371 | 19 | -31.1 | 4 | Shwachman-Bodian-Diamond syndrome protein homolog | / |

| | | | | | |
|---|---|---|---|---|---|
| miR-1190 | 24 | -35.6 | 4 | Sorcin | / |
| miR-1019* | 23 | -29.3 | 4 | Dynein light chain Tctex-type 1 (T-complex testis-specific protein 1 homolog) | / |
| miR-998 | 19 | -32.9 | 3 | / | / |
| miR-678 | 20 | -30.6 | 3 | / | / |
| miR-503 | 19 | -31.4 | 3 | V-type H+-transporting ATPase subunit F | [EC:3.6.3.14] |
| miR-466b-2* | 21 | -31.8 | 3 | ribosomal protein S8 | ko:K02993 small subunit ribosomal protein S7e |
| miR-4206-3p | 19 | -31.4 | 3 | hypothetical protein | / |
| miR-4194-3p | 20 | -30.8 | 3 | / | / |
| miR-4175-3p | 19 | -32.3 | 3 | U1 small nuclear ribonucleoprotein A | / |
| miR-4104-5p | 20 | -32.1 | 3 | / | / |
| miR-4088-3p | 18 | -26.5 | 3 | / | / |
| miR-4001b-5p | 21 | -29.6 | 3 | Ribosomal protein L37a | ko:K02922 large subunit ribosomal protein L37e |
| miR-3934 | 20 | -31.8 | 3 | Translocation-associated membrane protein 1 | / |
| miR-383 | 19 | -32 | 3 | / | / |
| miR-382 | 22 | -32.7 | 3 | / | / |
| miR-378c | 22 | -36.9 | 3 | protein KINase | [EC:2.7.11.1] ko:K02218 casein kinase 1 |
| miR-378 | 22 | -35.2 | 3 | kin-19 | [EC:2.7.11.1] ko:K02218 casein kinase 1 |
| miR-36c | 18 | -30.7 | 3 | heat shock protein 90kDa alpha | ko:K04079 molecular chaperone HtpG |
| miR-369-3p | 20 | -29.6 | 3 | eukaryotic translation initiation factor 3, subunit 3 gamma | ko:K03247 translation initiation factor eIF-3 subunit 3 |
| miR-3681 | 19 | -32.5 | 3 | carboxypeptidase N, polypeptide 1, 50kD | / |
| miR-3592 | 24 | -35 | 3 | Tubulin beta-2C chain | / |
| miR-3559-3p | 20 | -29.6 | 3 | ATPase | / |

| | | | | | |
|---|---|---|---|---|---|
| miR-3499 | 23 | -30.6 | 3 | / | / |
| miR-3497 | 19 | -32 | 3 | Cleavage and polyadenylation specificity factor subunit 4 | / |
| miR-331* | 18 | -30.4 | 3 | Centaurin-alpha 1 (Putative MAPK-activating protein PM25) | / |
| miR-27a | 21 | -31.3 | 3 | / | / |
| miR-2789 | 21 | -31.6 | 3 | / | / |
| miR-2772b | 19 | -28.2 | 3 | / | / |
| miR-2764 | 24 | -33.7 | 3 | / | / |
| miR-2463 | 20 | -31.4 | 3 | / | / |
| miR-219-5p | 23 | -28.9 | 3 | hypotheticial protein | / |
| miR-1957 | 20 | -26.3 | 3 | / | / |
| miR-190-5p | 23 | -33 | 3 | / | / |
| miR-18a | 24 | -34.7 | 3 | CTD small phosphatase-like protein | / |
| miR-181c | 23 | -33.9 | 3 | hypothetical protein | / |
| miR-181a | 23 | -34.2 | 3 | / | / |
| miR-1742 | 20 | -31.9 | 3 | Intersectin-1 (SH3 domain-containing protein 1A) | / |
| miR-1733 | 23 | -32.4 | 3 | / | / |
| miR-1725 | 22 | -27.6 | 3 | / | / |
| miR-151-5p | 21 | -29.9 | 3 | hypothetical protein | / |
| miR-150 | 23 | -33.1 | 3 | / | / |
| miR-148a | 22 | -28.9 | 3 | / | / |
| miR-1421ah* | 21 | -27.7 | 3 | / | / |
| miR-133c | 21 | -30.2 | 3 | / | / |
| miR-1293 | 22 | -28.9 | 3 | / | / |
| miR-124a | 19 | -30.5 | 3 | rps-26 | ko:K02976 small subunit ribosomal protein |

| | | | | | |
|---|---|---|---|---|---|
| | | | | | S26e |
| miR-124 | 19 | -28 | 3 | / | / |
| miR-9c | 20 | -29.7 | 2 | / | / |
| miR-96 | 20 | -28.8 | 2 | / | / |
| miR-93 | 23 | -29.9 | 2 | ribosomal protein, large P2 | ko:K02943 large subunit ribosomal protein LP2 |
| miR-733 | 20 | -29.3 | 2 | / | / |
| miR-654-5p | 19 | -30.9 | 2 | / | / |
| miR-61 | 23 | -33.4 | 2 | ITPKB | [EC:2.7.1.127] ko:K00911 1D-myo-inositol-triphosphate 3-kinase |
| miR-4162-5p | 26 | -31.6 | 2 | ribosomal protein, large P2 | ko:K02943 large subunit ribosomal protein LP2 |
| miR-4156-3p | 22 | -28.1 | 2 | / | / |
| miR-4154-3p | 19 | -30.4 | 2 | Mitochondrial 28S ribosomal protein S29 | / |
| miR-4082-3p | 18 | -30.5 | 2 | transcription initiation factor TFIIH subunit H1 | / |
| miR-4080-3p | 21 | -29.4 | 2 | CDK-activating kinase assembly factor MAT1 (RING finger protein MAT1) | / |
| miR-4077a-5p | 22 | -31.6 | 2 | / | / |
| miR-4055-5p | 20 | -28.7 | 2 | Proteinase inhibitor I2, Kunitz metazoa,domain-containing protein | / |
| miR-4044-5p | 19 | -27.6 | 2 | / | / |
| miR-4004-5p | 19 | -31.1 | 2 | Transmembrane 9 superfamily protein member 4 | / |
| miR-3915 | 21 | -30.2 | 2 | Sjoegren syndrome/scleroderma autoantigen 1 homolog | / |
| miR-3650 | 19 | -36.7 | 2 | Homeobox protein ceh-18 | / |
| miR-3271 | 23 | -29.6 | 2 | Guanine nucleotide-binding protein subunit beta 2-like 1 | / |

| | | | | | |
|---|---|---|---|---|---|
| miR-3203 | 22 | -32.6 | 2 | / | / |
| miR-3149 | 21 | -27.9 | 2 | hypothetical protein | / |
| miR-30a-5p | 24 | -31.4 | 2 | / | / |
| miR-2f | 19 | -26.3 | 2 | H/ACA ribonucleoprotein complex subunit 3 | / |
| miR-2d-3p | 24 | -32.2 | 2 | Uev1A | / |
| miR-2d-3p | 24 | -32.2 | 2 | putative ubiquitin-conjugating enzyme | / |
| miR-2c-3p | 29 | -36.8 | 2 | Sperm-associated antigen 6 | / |
| miR-2840 | 20 | -33.9 | 2 | U1 small nuclear ribonucleoprotein C | / |
| miR-2807c* | 22 | -28.8 | 2 | Major egg antigen (p40) | / |
| miR-24b | 21 | -32.6 | 2 | / | / |
| miR-2178 | 20 | -31 | 2 | / | / |
| miR-21 | 23 | -31.2 | 2 | / | / |
| miR-2014 | 25 | -37.1 | 2 | / | / |
| miR-200a* | 22 | -29.8 | 2 | / | / |
| miR-1948* | 21 | -28.5 | 2 | U6 snRNA-associated Sm-like protein LSm5 | / |
| miR-191 | 23 | -32 | 2 | Tctex1 domain containing-protein 1 | / |
| miR-145* | 23 | -31.2 | 2 | mitochondrial ribosomal protein L43 | / |
| miR-144* | 23 | -31.9 | 2 | Serine/threonine-protein phosphatase 4 catalytic subunit | [EC:3.1.3.16] |
| miR-143 | 20 | -28 | 2 | / | / |
| miR-1421ac | 25 | -35.4 | 2 | Calcium-binding EF-hand,domain-containing protein | / |
| miR-141 | 23 | -30.2 | 2 | / | / |
| miR-140* | 19 | -27.1 | 2 | hypotheticial protein | / |
| miR-135b | 23 | -32.2 | 2 | hypothetical protein | / |
| miR-128 | 22 | -29.7 | 2 | Suppressor of profilin 2 | ko:K05757 actin related protein 2/3 complex, subunit 1A/1B, 41kDa |
| miR-1253 | 19 | -29.7 | 2 | hypotheticial protein | / |

| | | | | | |
|---|---|---|---|---|---|
| miR-122b | 25 | -34.2 | 2 | Universal stress protein | / |
| miR-122 | 24 | -30.7 | 2 | / | / |
| miR-98 | 23 | -31.4 | 1 | hypotheticial protein | / |
| miR-891a | 20 | -32 | 1 | kin-19 | [EC:2.7.11.1] ko:K02218 casein kinase 1 |
| miR-882 | 22 | -33.9 | 1 | / | / |
| miR-7b | 23 | -32.3 | 1 | Ribosome biogenesis regulatory protein homolog | / |
| miR-753b-5p | 21 | -29.9 | 1 | Immunogenic miracidial antigen 8I' | / |
| miR-750 | 22 | -33 | 1 | H+-transporting ATPase | / |
| miR-745a | 20 | -31.6 | 1 | / | / |
| miR-71c | 22 | -31.5 | 1 | / | / |
| miR-71b-5p | 23 | -32.3 | 1 | hypothetical protein | / |
| miR-7 | 24 | -32.3 | 1 | Ribosome biogenesis regulatory protein homolog | / |
| miR-548p | 23 | -29.2 | 1 | / | / |
| miR-548f | 21 | -29.8 | 1 | NEDD8 precursor | / |
| miR-520c-5p | 24 | -32.6 | 1 | hypothetical protein | / |
| miR-488 | 19 | -25.2 | 1 | / | / |
| miR-452 | 24 | -27.5 | 1 | / | / |
| miR-4272 | 20 | -26 | 1 | hypotheticial protein | / |
| miR-4219-5p | 19 | -26.4 | 1 | / | / |
| miR-4214-5p | 20 | -30.5 | 1 | / | / |
| miR-421 | 20 | -29.3 | 1 | / | / |
| miR-4145-3p | 18 | -31.1 | 1 | / | / |
| miR-4126-5p | 22 | -28 | 1 | / | / |
| miR-4120-5p | 18 | -25.3 | 1 | hypotheticial protein | / |
| miR-4090-3p | 19 | -27.4 | 1 | / | / |
| miR-4032-5p | 20 | -27.7 | 1 | hypothetical protein | / |

| | | | | | |
|---|---|---|---|---|---|
| miR-4006f-5p | 20 | -26.7 | 1 | / | / |
| miR-4006b-5p | 22 | -27.1 | 1 | / | / |
| miR-4003a-3p | 21 | -26.8 | 1 | hypotheticial protein | / |
| miR-4001h-5p | 21 | -27.6 | 1 | hypotheticial protein | / |
| miR-36a | 22 | -31.2 | 1 | Immunogenic miracidial antigen 8I' | / |
| miR-3668 | 21 | -26.5 | 1 | ATPase, F0 complex, subunit E, mitochondrial,domain-containing protein | / |
| miR-36-5p | 23 | -34.5 | 1 | hypothetical protein | / |
| miR-3654 | 21 | -29.8 | 1 | / | / |
| miR-3558-3p | 20 | -31.7 | 1 | / | / |
| miR-3526 | 20 | -26.8 | 1 | / | / |
| miR-3495 | 20 | -28.4 | 1 | / | / |
| miR-3493 | 21 | -31.7 | 1 | / | / |
| miR-327 | 19 | -29.7 | 1 | DNA replication licensing factor MCM2 (Minichromosome maintenance protein 2 homolog) | / |
| miR-31-5p | 23 | -35.1 | 1 | hypothetical protein | / |
| miR-3148 | 24 | -27.3 | 1 | / | / |
| miR-3125 | 19 | -27 | 1 | / | / |
| miR-3115 | 18 | -25.6 | 1 | / | / |
| miR-3107* | 18 | -28.5 | 1 | hypothetical protein | / |
| miR-30c | 21 | -27.3 | 1 | / | / |
| miR-30a | 23 | -29.1 | 1 | / | / |
| miR-3080-5p | 19 | -29.3 | 1 | Peptidase C2, calpain,domain-containing protein | / |
| miR-308 | 20 | -30.9 | 1 | TRK-fused protein | ko:K09292 protein TFG |
| miR-3063* | 23 | -33 | 1 | / | / |
| miR-3002 | 20 | -30.7 | 1 | Rab8 | ko:K07976 Rab family, other |

| | | | | | |
|---|---|---|---|---|---|
| miR-2e-5p | 22 | -28.6 | 1 | hypotheticial protein | / |
| miR-2b-5p | 22 | -27.2 | 1 | Histone H2A | / |
| miR-2989 | 18 | -28.7 | 1 | / | / |
| miR-2917 | 18 | -29 | 1 | / | / |
| miR-2812 | 20 | -29.2 | 1 | / | / |
| miR-27d | 21 | -31.8 | 1 | / | / |
| miR-27b | 21 | -31.2 | 1 | / | / |
| miR-279b* | 18 | -27.9 | 1 | / | / |
| miR-277b-3p | 20 | -29.5 | 1 | Translocon-associated protein subunit alpha precursor | / |
| miR-2755 | 22 | -29.8 | 1 | / | / |
| miR-2703* | 23 | -32 | 1 | / | / |
| miR-26a | 22 | -25.9 | 1 | Histone H2A | / |
| miR-2696 | 24 | -28 | 1 | / | / |
| miR-254 | 24 | -27.9 | 1 | hypothetical protein | / |
| miR-2479 | 19 | -25.9 | 1 | / | / |
| miR-2444 | 22 | -28.4 | 1 | Egg protein CP111 | / |
| miR-2437 | 21 | -27.9 | 1 | / | / |
| miR-2434 | 23 | -28.1 | 1 | 26 proteasome complex subunit DSS1 | / |
| miR-2421 | 22 | -29.3 | 1 | / | / |
| miR-240 | 25 | -30.4 | 1 | / | / |
| miR-2318 | 22 | -25.7 | 1 | / | / |
| miR-2304 | 20 | -30.4 | 1 | / | / |
| miR-2238b | 18 | -27 | 1 | Saposin B domain-containing protein | / |
| miR-2235 | 21 | -25.9 | 1 | / | / |
| miR-222a* | 19 | -29.4 | 1 | / | / |
| miR-2206-3p | 18 | -30 | 1 | Qprt | [EC:2.4.2.19] ko:K00767 |

| | | | | | |
|---|---|---|---|---|---|
| | | | | | nicotinate-nucleotide pyrophosphorylase (carboxylating) |
| miR-214* | 19 | -28.1 | 1 | / | / |
| miR-20a-2* | 22 | -28.8 | 1 | hypotheticial protein | / |
| miR-200c | 23 | -28.3 | 1 | / | / |
| miR-1b | 23 | -28.4 | 1 | / | / |
| miR-1a | 24 | -29.5 | 1 | / | / |
| miR-196b | 21 | -29.5 | 1 | / | / |
| miR-190b | 24 | -32.1 | 1 | / | / |
| miR-190-3p | 22 | -29.9 | 1 | ribosomal protein S12 | ko:K02951 small subunit ribosomal protein S12e |
| miR-186 | 23 | -32.7 | 1 | / | / |
| miR-183 | 23 | -29.7 | 1 | hypotheticial protein | / |
| miR-1814 | 20 | -28.8 | 1 | 26 proteasome complex subunit DSS1 | / |
| miR-1757 | 21 | -29.2 | 1 | roadblock | ko:K01509 adenosinetriphosphatase |
| miR-1738 | 23 | -39.7 | 1 | Sad1/unc-84-like protein 2 | / |
| miR-1700 | 23 | -30.6 | 1 | / | / |
| miR-17 | 23 | -28.5 | 1 | hypotheticial protein | / |
| miR-1689 | 20 | -35.1 | 1 | / | / |
| miR-1600 | 20 | -28.1 | 1 | hypothetical protein | / |
| miR-1568 | 18 | -35 | 1 | Sad1/unc-84-like protein 2 | / |
| miR-155* | 20 | -27.7 | 1 | / | / |
| miR-152 | 21 | -29.6 | 1 | Cupin, RmlC-type,domain-containing protein | / |
| miR-1422i | 20 | -31.1 | 1 | Cell differentiation protein rcd1 | / |
| miR-1422e | 25 | -35.4 | 1 | Alpha-taxilin | / |
| miR-1421af* | 19 | -30.8 | 1 | Cathepsin B-like cysteine proteinase precursor | [EC:3.4.22.-] |

| | | | | | |
|---|---|---|---|---|---|
| miR-140 | 22 | -29.8 | 1 | Peptidase C2, calpain,domain-containing protein | / |
| miR-1370 | 18 | -28.1 | 1 | / | / |
| miR-1358 | 20 | -32.9 | 1 | hypothetical protein | / |
| miR-1357 | 19 | -25 | 1 | / | / |
| miR-1339* | 26 | -29.9 | 1 | hypotheticial protein | / |
| miR-130b | 23 | -33.1 | 1 | / | / |
| miR-1278 | 22 | -29.9 | 1 | SAM domain-containing protein | / |
| miR-126-5p | 19 | -30.2 | 1 | / | / |
| miR-126-3p | 22 | -31.2 | 1 | / | / |
| miR-10c | 22 | -29.8 | 1 | proteasome (prosome, macropain) subunit, beta type, 6 | [EC:3.4.25.1] ko:K02738 20S proteasome subunit beta 1 |
| miR-101a | 22 | -33.2 | 1 | putative protein affecting Mg2+/Co2+ transport | ko:K06195 ApaG protein |
| miR-101 | 21 | -31.5 | 1 | putative protein affecting Mg2+/Co2+ transport | ko:K06195 ApaG protein |
| lin-4 | 20 | -31 | 1 | Caltractin (Centrin) | / |
| let-7f | 22 | -28.1 | 1 | / | / |
