# Supplementary material for: Characterization of MicroRNAs from Orientobilharzia turkestanicum, a Neglected Blood Fluke of Human and Animal Health Significance
Source: PLoS One. 2012 Oct 10;7(10):e47001. doi: 10.1371/journal.pone.0047001 (PMC3468544; doi:10.1371/journal.pone.0047001)
Supplement: Figure S2 — Complementary Structure of miRNA with their star sequences. (PDF) [file pone.0047001.s002.pdf]

Additional file 2. Complementary Structure of miRNA with their star sequences

Name	Target	Mfe (kcal/mol)	P-value	Complementary Structure
**NADH dehydrogenase**				
miR-4144-3p	SJFCE3378.004	-30.2	0.00372	target 5' G G 3'<br>CUGGUGAUGCAAGAUU<br>GACUACUACGUUCUGA<br>miRNA 3' G A 5'
miR-1837	SJFCE2420.002	-35.4	0.001062	target 5' C U C C 3'<br>UG GUCAACAGUCCCU CUCA<br>AC CAGUUGUCAGGGA GAGU<br>miRNA 3' UCUU CU 5'
miR-125b*	SJFCA2649.001	-33.2	0.003403	target 5' A U G 3'<br>CCCAAGAGCUUG CUUG<br>GGGUUCUCGGAC GAAC<br>miRNA 3' CCA U A 5'
miR-36a*	SJFCE3371.004	-30.2	0.008135	target 5' C C U 3'<br>GAACCAGGUACAAGA AUUCA<br>UUUGGUUCAUGUUCU UAAGU<br>miRNA 3' 5'

**ATPase**				
miR-3666	SJFCE5459	-30.6	0.002343	target 5' G G G 3'<br>CGCGCGUUU CGUGUGUG UGCU<br>GCGCGUAGA GUGUACGU ACGG<br>miRNA 3' A U G U 5'
miR-4115-5p	SJFCE3770.010	-30	0.009561	target 5' U C C 3'<br>UGG GAGUGCAGACCA<br>GCC CUCAUGUCUGGU<br>miRNA 3' UUUCUU U 5'
miR-4038-3p	SJFCE3300.005	-27.9	0.003849	target 5' G A A 3'<br>AGAUUAACCGAGU CAAUG<br>UCUAGUUGGUUCA GUUAC<br>miRNA 3' C 5'
miR-3668	SJFCE1459	-26.5	0.008551	target 5' G G A 3'<br>UUUACGGA CUCUGCGUUU<br>AAAUGUCU GAGAUGUAAA<br>miRNA 3' UA A 5'
miR-3559-3p	SJFCE2856.001	-29.6	0.005681	target 5' A C G 3'<br>UGAUAUGACUCAGUUU CU<br>GCUGUACUGAGUCAGA GA<br>miRNA 3' G U 5'

miR-503	SJFCE2163.001	-31.4	0.001014	target 5’ G C 3’<br>GCUGUUUCCGCUGC<br>UGACAAGGGCGACG<br>miRNA 3’ AAA AU 5’
**Transcription initiation factor**				
miR-369-3p	SJFCA3197.003	-29.6	0.008818	target 5’ U A 3’<br>UGUCAACGGUGUGUUAUU<br>ACAGUUGCUACAUAAUAA<br>miRNA 3’ UU 5’
miR-139	SJFCA3498.001	-32.6	0.001443	target 5’ A U A 3’<br>GGGACAU UGCACUGGA<br>CCUUGUG ACGUGACCU<br>miRNA 3’ A U C 5’
miR-4082-3p	SJFCE1620	-30.5	0.008156	target 5’ A C 3’<br>AGUUCAGCUCCAACUG<br>UCAAGUUGAGGUUGGC<br>miRNA 3’ AA 5’
miR-4148-5p	SJFCA0288	-33.6	0.002135	target 5’ U A G 3’<br>GUGU CCGGUGCACG CUCU<br>CACA GGUCACGUGC GAGA<br>miRNA 3’ U G 5’

Splicing factor				
miR-23a*	SJFCA0598	-31.4	0.008199	target 5' A G A 3'<br>UG GUCGCCAGAAAUCC<br>GC UAGUGGUCUUUAGG<br>miRNA 3' UUUCA G 5'
miR-767-3p	SJFCE3755.003	-33.4	0.005437	target 5' A C G 3'<br>GAAACCAU GGGUA GUGGUGGU<br>UUUUGGUA CCCGU CAUCAUCG<br>miRNA 3' C A U 5'
miR-463	SJFCE3755.003	-30.5	0.00905	target 5' G A 3'<br>ACCCGGUUCUCGUCG<br>UGGGUUAAGAGCAGU<br>miRNA 3' GA AGU 5'
miR-598	SJFCE3755.014	-30	0.004638	target 5' A A 3'<br>UGGCGGUGGACGUGAC<br>ACUGCUAUUUGCACUG<br>miRNA 3' CUAU 5'
miR-2881	SJFCA0344	-43.3	0.000057	target 5' C C 3'<br>CCACCACCUCCGCCCC<br>GGUGGUGGGGGCGGGG<br>miRNA 3' GUA U 5'

**Signal transduction**				
miR-532-3p	SJFCE3560.007	-35.9	0.002175	target 5' A C C A U 3'<br>GCAAGCC UGGGU G GG GAGG<br>CGUUCGG ACCCA C CC CUCC<br>miRNA 3' A A A 5'
miR-236	SJFCE3743.002	-31.7	0.003428	target 5' C C A A 3'<br>AUCCUCGUU UCGA CAGUAUUG<br>UAGGAGUAA AGCU GUCAUAAU<br>miRNA 3' A U A 5'
**Growth factor receptor**				
miR-1942	SJFCA2712.001	-31.7	0.008911	target 5' A U 3'<br>ACCGGAUGGGGCAUUCG<br>UGGUCUACUCUGUAAGU<br>miRNA 3' GUA 5'
miR-71a	SJFCA3391.005	-35	0.008505	target 5' A C 3'<br>UUCAUCUUACUACUAGUGUUU<br>AAGUAGAGUGAUGGUCGCAGA<br>miRNA 3' AAGU 5'
**Zinc finger protein**				
miR-4020b-5p	SJFCA0201	-33.6	0.001724	target 5' C A 3'<br>UAUGUACCGCC ACCACC<br>GUACGUGGUGG UGGUGG<br>miRNA 3' U 5'

miR-92e-5p	SJFCA0201	-36.2	0.00102	target 5' C A 3'<br>CUAUGUACCGCC ACCACC<br>GGUACGUGGUGG UGGUGG<br>miRNA 3' U C 5'
miR-252	SJFCA0201	-36.8	0.000521	target 5' C C 3'<br>CCUGCGGCACCAGUUC<br>GGGCGUCGUGGUCAAG<br>miRNA 3' A AC 5'
miR-4150-5p	SJFCA0510	-35.7	0.000999	target 5' G G 3'<br>UCACAGUCGUAUAGUUAUC<br>AGUGUCAGCGUAUCGAUAG<br>miRNA 3' 5'
miR-4101-3p	SJFCE3594.008	-28.5	0.007164	target 5' C G G 3'<br>UCCA AUAUACUACGUUCA<br>GGGU UAUAUGAUGUAGGU<br>miRNA 3' G 5'
**Egg protein**				
miR-320c	SJFCE2139.001	-31.1	0.006281	target 5' U A 3'<br>CCCUUUCAAUCCGGC<br>GGGAGAGUUGGGUCG<br>miRNA 3' AAAA 5'

miR-2444	SJFCE3676.010	-28.4	0.009058	target 5' U U C 3'<br>GAACAAAUAAC ACAGGACAAA<br>UUUGUUUGUUG UGUUCUGUUU<br>miRNA 3' U 5'
miR-2807c*	SJFCE3845.025	-28.8	0.008918	target 5' A U G 3'<br>GGUC GACGGUGUUGGAUGAUG<br>UCAG UUGUUACGGUUUACUAC<br>miRNA 3' G 5'
**Heat shock protein**				
miR-36c	SJFCE2899.003	-30.7	0.005237	target 5' G C G 3'<br>GAGU GUCUGCC GGUGG<br>CUUA CAGACGG CCACC<br>miRNA 3' A G 5'
miR-486-5p	SJFCE2701.002	-31.8	0.007241	target 5' A U A C 3'<br>UUGGGGC A G UUAGUACAGGA<br>AGCCCCG U C AGUCAUGUCCU<br>miRNA 3' G 5'
miR-669g	SJFCA3842.001	-32.1	0.001205	target 5' U G 3'<br>GUAGUUGCACACGGUCCAG<br>UAUCAGUGUGUGUUAGGUU<br>miRNA 3' ACGU 5'
